# Phenyl 4,6-di-*O*-acetyl-2,3-dide­oxy-1-thio-α-d-*erythro*-hex-2-enopyran­oside

**DOI:** 10.1107/S1600536811040165

**Published:** 2011-10-05

**Authors:** Henok H. Kinfe, Fanuel M. Mebrahtu, Alfred Muller

**Affiliations:** a Research Center for Synthesis and Catalysis, Department of Chemistry, University of Johannesburg (APK Campus), PO Box 524, Auckland Park, Johannesburg 2006, South Africa

## Abstract

The pyranosyl ring in the title compound, C_16_H_18_O_5_S, adopts an envelope conformation, with the acetyl groups in equatorial positions. In the crystal, weak C—H⋯O inter­actions link the molecules into chains.

## Related literature

For details of the Ferrier arrangement, see: Ferrier & Prasad (1969 ▶). For the synthesis of pseudoglycals utilizing the Ferrier arrangement, see: López *et al.* (1995 ▶); Yadav *et al.* (2001 ▶). For applications of pseudoglycals, see: Domon *et al.* (2005 ▶); Danishefsky & Bilodeau (1996 ▶); Griffith & Danishefsky (1991 ▶); Halcomb *et al.* (1995 ▶); Bracherro *et al.* (1998 ▶); Dorgan & Jackson (1996 ▶); Chambers *et al.* (2005 ▶); Minuth & Boysen (2009 ▶). For background to the synthetic methodology of glycosides, see: Kinfe *et al.* (2011 ▶). For the preparation of the acid catalyst NaHSO_4_-SiO_2_, see: Breton (1997 ▶). For ring puckering analysis see, Cremer & Pople (1975 ▶). For a description of the Csambridge Structural Database, see: Allen (2002 ▶).
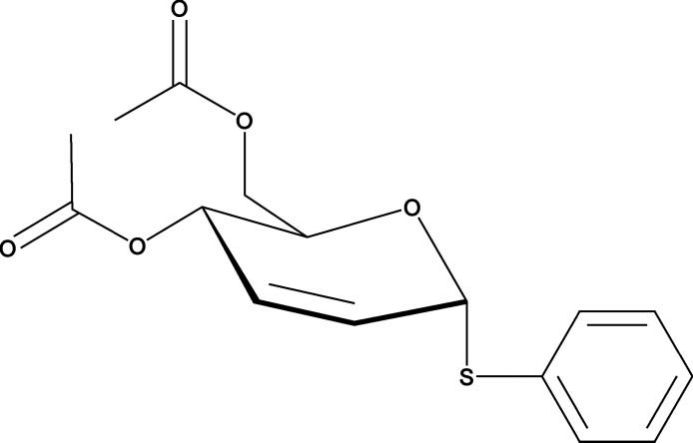


## Experimental

### 

#### Crystal data

C_16_H_18_O_5_S
*M* *_r_* = 322.36
Monoclinic, 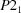

*a* = 5.2330 (4) Å
*b* = 13.470 (1) Å
*c* = 11.1760 (9) Å
β = 97.291 (2)°
*V* = 781.41 (10) Å^3^
*Z* = 2
Mo *K*α radiation
μ = 0.23 mm^−1^
*T* = 100 K
0.42 × 0.37 × 0.27 mm

#### Data collection

Bruker APEXII DUO 4K KappaCCD diffractometer
Absorption correction: multi-scan (*SADABS*; Bruker, 2008 ▶) *T*_min_ = 0.910, *T*_max_ = 0.941
10609 measured reflections
3839 independent reflections
3771 reflections with *I* > 2σ(*I*)
*R*_int_ = 0.020

#### Refinement

*R*[*F* ^2^ > 2σ(*F* ^2^)] = 0.025
*wR*(*F* ^2^) = 0.066
*S* = 1.06
3839 reflections
201 parameters
1 restraint
H-atom parameters constrained
Δρ_max_ = 0.31 e Å^−3^
Δρ_min_ = −0.20 e Å^−3^
Absolute structure: Flack (1983 ▶), 1824 Friedel pairs
Flack parameter: 0.04 (4)

### 

Data collection: *APEX2* (Bruker, 2011 ▶); cell refinement: *SAINT* (Bruker, 2008 ▶); data reduction: *SAINT* and *XPREP* (Bruker, 2008 ▶); program(s) used to solve structure: *SIR97* (Altomare *et al.*, 1999 ▶); program(s) used to refine structure: *SHELXL97* (Sheldrick, 2008 ▶); molecular graphics: *DIAMOND* (Brandenburg & Putz, 2005 ▶); software used to prepare material for publication: *WinGX* (Farrugia, 1999 ▶).

## Supplementary Material

Crystal structure: contains datablock(s) global, I. DOI: 10.1107/S1600536811040165/fj2451sup1.cif

Structure factors: contains datablock(s) I. DOI: 10.1107/S1600536811040165/fj2451Isup2.hkl

Additional supplementary materials:  crystallographic information; 3D view; checkCIF report

## Figures and Tables

**Table 1:** Hydrogen-bond geometry (Å, °)

*D*—H⋯*A*	*D*—H	H⋯*A*	*D*⋯*A*	*D*—H⋯*A*
C13—H13*B*⋯O5^i^	0.98	2.44	3.3506 (15)	154

Symmetry code: (i) 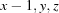.

**Fig. 1.**
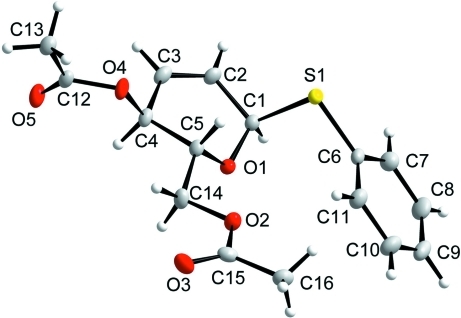
View of (I). Displacement ellipsoids are drawn at a 50% probability level.

## supplementary crystallographic information

### Comment

Glycals, 1,2-unsaturated pyranoses, undergo acid catalyzed allylic rearrangement in the presence of alcohols to provide 2,3-unsaturated glycosides (pseudoglycals) (see Ferrier & Prasad, 1969). This reaction is referred as the Ferrier rearrangement reaction. Since the reaction proceeds *via* an oxycarbonium intermediate, thiols, halides and other nucleophiles can be employed besides alcohols to produce corresponding glycosides (see López *et al.*, 1995; Yadav *et al.*, 2001). The pseudoglycal products from the Ferrier rearrangement reaction have been used as chiral building blocks in the synthesis of antibiotics (see Domon *et al.*, 2005), oligosaccharides (see Danishefsky & Bilodeau, 1996; Griffith & Danishefsky, 1991; Halcomb *et al.*, 1995), nucleosides (see Bracherro *et al.*, 1998), glycopeptides (see Dorgan & Jackson, 1996; Chambers *et al.*, 2005) and also as chiral ligands in asymmetric synthesis (see Minuth & Boysen, 2009). Among other thioglycosides, phenyl 2,3-unsaturated thioglycosides have been extensively employed in organic synthesis such as in the elegant total synthesis of allosamidin (chitinase inhibitor), esperamicin and Calicheamicin (see Danishefsky & Bilodeau, 1996; Griffith & Danishefsky, 1991; Halcomb *et al.*, 1995). Due to the importance of this type of thioglycosides, herein we report the structural analysis of phenyl 2,3-unsaturated thioglycoside I.

The title compound (see Fig. 1, scheme 1) crystallizes in the *P*2_1_ (*Z*=2) space group resulting in molecules lying on general positions in the unit cell. All bond lengths are within their normal ranges (Allen, 2002) with the acetyl groups all in equatorial positions. The pyran ring is in an envelope conformation with ring puckering parameters of q_2_ = 0.4212 (12) Å, q_3_ = 0.2974 (12) Å, Q = 0.5156 (11) Å and φ_2_ = 321.05 (17)° (see Cremer & Pople, 1975). Weak C—H···O/S interactions (see Table 1) stabilize the crystal structure.

### Experimental

To a solution of a tri-*O*-acetyl-D-glucal (100 mg, 0.36 mmol) in CH_3_CN (1 ml) NaHSO_4_-SiO_2_ (2.5 mg, 3.0 mmol NaHSO_4_/g) was added (see Breton, 1997). The resulting mixture was stirred at 80 °C for 5 min. After adding silica gel to the reaction mixture at room temperature, the solvent was evaporated *in vacuo* without heating until a free-flowing solid was obtained. The resulting solid was column chromatographed using 1:9 ethyl acetate:hexane eluent to afford α:β (4:1) mixture of 2,3-unsaturated glycosides in 96% yield as a white solid (see Kinfe *et al.*, 2011). Recrystalization from a mixture of DCM and hexane afforded the title thioglycoside I in 60% yield as white crystals. Analytical data: ^1^H NMR (CDCl_3_, 300 MHz): δ 7.51 (d, *J* = 7.2 Hz, 2H), 7.29–7.17 (m, 3H), 6.03 (d, *J* = 10.2 Hz, 1H), 5.83 (d, *J* = 10.8 Hz, 1H), 5.73 (s, 1H), 5.35 (d, *J* = 9.6 Hz, 1H), 4.60–4.13 (m, 3H), 2.07 (s, 3H), 2.03 (s, 3H); ^13^C NMR (CDCl_3_, 75 MHz): δ 170.7, 170.2, 134.7, 131.7, 128.9, 128.5, 127.6, 83.6, 67.2, 65.0, 63.0, 20.9, 20.7.

### Refinement

All hydrogen atoms were positioned in geometrically idealized positions with C—H = 1.00 Å, 0.99 Å, 0.98 Å and 0.95 Å for methine, methylene, methyl and aromatic H atoms respectively. All hydrogen atoms were allowed to ride on their parent atoms with *U*_iso_(H) = 1.2*U*_eq_, except for methyl where *U*_iso_(H) = 1.5*U*_eq_ was utilized. The initial positions of methyl hydrogen atoms were located from a Fourier difference map and refined as fixed rotor. The D enantiomer refined to a final Flack parameter of 0.04 (4). The highest residual electron density of 0.31 e.Å^-3^ is 0.88 Å from S1 representing no physical meaning.

### Crystal data

**Table:**

C_16_H_18_O_5_S	*F*(000) = 340
*M**_r_* = 322.36	*D*_x_ = 1.37 Mg m^−^^3^
Monoclinic, *P*2_1_	Mo *K*α radiation, λ = 0.71073 Å
Hall symbol: P 2yb	Cell parameters from 7987 reflections
*a* = 5.2330 (4) Å	θ = 3.0–28.3°
*b* = 13.470 (1) Å	µ = 0.23 mm^−^^1^
*c* = 11.1760 (9) Å	*T* = 100 K
β = 97.291 (2)°	Prism, colourless
*V* = 781.41 (10) Å^3^	0.42 × 0.37 × 0.27 mm
*Z* = 2	

### Data collection

**Table:**

Bruker APEXII DUO 4K KappaCCD diffractometer	3839 independent reflections
graphite	3771 reflections with *I* > 2σ(*I*)
Detector resolution: 8.4 pixels mm^-1^	*R*_int_ = 0.020
φ and ω scans	θ_max_ = 28.4°, θ_min_ = 1.8°
Absorption correction: multi-scan (*SADABS*; Bruker, 2008)	*h* = −6→6
*T*_min_ = 0.910, *T*_max_ = 0.941	*k* = −17→17
10609 measured reflections	*l* = −14→14

### Refinement

**Table:**

Refinement on *F*^2^	Secondary atom site location: difference Fourier map
Least-squares matrix: full	Hydrogen site location: inferred from neighbouring sites
*R*[*F*^2^ > 2σ(*F*^2^)] = 0.025	H-atom parameters constrained
*wR*(*F*^2^) = 0.066	*w* = 1/[σ^2^(*F*_o_^2^) + (0.0412*P*)^2^ + 0.0965*P*] where *P* = (*F*_o_^2^ + 2*F*_c_^2^)/3
*S* = 1.06	(Δ/σ)_max_ = 0.001
3839 reflections	Δρ_max_ = 0.31 e Å^−^^3^
201 parameters	Δρ_min_ = −0.20 e Å^−^^3^
1 restraint	Absolute structure: Flack (1983), 1824 Friedel pairs
Primary atom site location: structure-invariant direct methods	Flack parameter: 0.04 (4)

### Special details

**Table:**

Experimental. The intensity data was collected on a Bruker *APEX* Duo 4 K KappaCCD diffractometer using an exposure time of 10 s/frame. A total of 1490 frames were collected with a frame width of 0.5° covering up to θ = 28.36° with 99.8% completeness accomplished.
Geometry. All e.s.d.'s (except the e.s.d. in the dihedral angle between two l.s. planes) are estimated using the full covariance matrix. The cell e.s.d.'s are taken into account individually in the estimation of e.s.d.'s in distances, angles and torsion angles; correlations between e.s.d.'s in cell parameters are only used when they are defined by crystal symmetry. An approximate (isotropic) treatment of cell e.s.d.'s is used for estimating e.s.d.'s involving l.s. planes.
Refinement. Refinement of *F*^2^ against ALL reflections. The weighted *R*-factor *wR* and goodness of fit *S* are based on *F*^2^, conventional *R*-factors *R* are based on *F*, with *F* set to zero for negative *F*^2^. The threshold expression of *F*^2^ > σ(*F*^2^) is used only for calculating *R*-factors(gt) *etc*. and is not relevant to the choice of reflections for refinement. *R*-factors based on *F*^2^ are statistically about twice as large as those based on *F*, and *R*- factors based on ALL data will be even larger.

### Fractional atomic coordinates and isotropic or equivalent isotropic displacement parameters (Å^2^)

**Table:**

	*x*	*y*	*z*	*U*_iso_*/*U*_eq_	
S1	0.97008 (5)	0.73210 (2)	0.55390 (2)	0.01758 (7)	
O1	1.10976 (16)	0.64668 (6)	0.77149 (7)	0.01499 (16)	
O2	0.67619 (16)	0.67526 (6)	0.89081 (8)	0.01858 (18)	
O3	0.43919 (18)	0.61581 (8)	1.02918 (8)	0.0242 (2)	
O4	0.82603 (16)	0.40209 (6)	0.72849 (8)	0.01964 (18)	
O5	1.07925 (17)	0.28011 (7)	0.81406 (9)	0.02305 (19)	
C1	1.1974 (2)	0.65826 (9)	0.65814 (10)	0.0151 (2)	
H1	1.3636	0.6957	0.6713	0.018*	
C2	1.2504 (2)	0.56087 (10)	0.60152 (11)	0.0181 (2)	
H2	1.3308	0.5601	0.5299	0.022*	
C3	1.1873 (2)	0.47589 (9)	0.64935 (12)	0.0193 (2)	
H3	1.2388	0.4154	0.6158	0.023*	
C4	1.0364 (2)	0.47220 (9)	0.75525 (12)	0.0167 (2)	
H4	1.1499	0.4524	0.8302	0.02*	
C5	0.9137 (2)	0.57276 (9)	0.77150 (10)	0.0153 (2)	
H5	0.7744	0.5852	0.7033	0.018*	
C6	1.0169 (2)	0.84785 (9)	0.63084 (11)	0.0169 (2)	
C7	1.2088 (3)	0.91243 (10)	0.60335 (13)	0.0234 (3)	
H7	1.313	0.8954	0.5427	0.028*	
C8	1.2472 (3)	1.00164 (11)	0.66480 (14)	0.0282 (3)	
H8	1.3785	1.0457	0.6463	0.034*	
C9	1.0957 (3)	1.02695 (10)	0.75292 (14)	0.0264 (3)	
H9	1.123	1.0882	0.7948	0.032*	
C10	0.9041 (3)	0.96298 (11)	0.77997 (13)	0.0274 (3)	
H10	0.7995	0.9805	0.8402	0.033*	
C11	0.8644 (2)	0.87300 (10)	0.71901 (13)	0.0228 (3)	
H11	0.7333	0.829	0.7378	0.027*	
C12	0.8777 (2)	0.30606 (9)	0.76035 (11)	0.0163 (2)	
C13	0.6527 (2)	0.24055 (10)	0.71904 (11)	0.0195 (2)	
H13A	0.6456	0.2284	0.6322	0.029*	
H13B	0.4933	0.2731	0.7354	0.029*	
H13C	0.6719	0.1772	0.7624	0.029*	
C14	0.8047 (2)	0.58030 (9)	0.88967 (11)	0.0196 (2)	
H14A	0.681	0.5257	0.897	0.024*	
H14B	0.9446	0.5758	0.958	0.024*	
C15	0.4963 (2)	0.68315 (10)	0.96714 (11)	0.0192 (2)	
C16	0.3881 (3)	0.78618 (10)	0.96492 (12)	0.0234 (3)	
H16A	0.2147	0.7843	0.9892	0.035*	
H16B	0.3795	0.8133	0.8831	0.035*	
H16C	0.4993	0.8283	1.021	0.035*	

### Atomic displacement parameters (Å^2^)

**Table:**

	*U*^11^	*U*^22^	*U*^33^	*U*^12^	*U*^13^	*U*^23^
S1	0.02171 (13)	0.01391 (12)	0.01645 (12)	0.00008 (11)	−0.00018 (9)	−0.00028 (11)
O1	0.0158 (4)	0.0132 (4)	0.0160 (4)	−0.0031 (3)	0.0023 (3)	−0.0005 (3)
O2	0.0180 (4)	0.0171 (4)	0.0219 (4)	0.0008 (3)	0.0073 (3)	0.0004 (3)
O3	0.0226 (4)	0.0306 (5)	0.0202 (4)	0.0000 (4)	0.0058 (3)	0.0053 (4)
O4	0.0152 (4)	0.0108 (4)	0.0315 (5)	−0.0018 (3)	−0.0023 (3)	0.0026 (3)
O5	0.0170 (4)	0.0158 (4)	0.0359 (5)	0.0016 (3)	0.0017 (4)	0.0043 (4)
C1	0.0144 (5)	0.0133 (5)	0.0176 (5)	0.0004 (4)	0.0019 (4)	−0.0003 (4)
C2	0.0163 (5)	0.0162 (5)	0.0224 (6)	0.0024 (4)	0.0045 (4)	−0.0037 (5)
C3	0.0150 (5)	0.0155 (6)	0.0272 (6)	0.0016 (4)	0.0017 (5)	−0.0048 (5)
C4	0.0138 (5)	0.0110 (5)	0.0246 (6)	−0.0011 (4)	−0.0002 (4)	0.0007 (4)
C5	0.0133 (5)	0.0129 (5)	0.0193 (5)	−0.0018 (4)	0.0009 (4)	0.0004 (4)
C6	0.0195 (5)	0.0122 (5)	0.0179 (5)	0.0013 (4)	−0.0019 (4)	0.0008 (4)
C7	0.0238 (6)	0.0216 (7)	0.0251 (6)	−0.0026 (5)	0.0036 (5)	0.0000 (5)
C8	0.0287 (7)	0.0194 (6)	0.0356 (8)	−0.0069 (5)	0.0003 (6)	−0.0004 (5)
C9	0.0284 (7)	0.0155 (6)	0.0324 (7)	0.0036 (5)	−0.0072 (5)	−0.0040 (5)
C10	0.0278 (7)	0.0223 (6)	0.0320 (7)	0.0045 (5)	0.0039 (5)	−0.0075 (5)
C11	0.0222 (6)	0.0174 (6)	0.0291 (7)	0.0003 (5)	0.0041 (5)	−0.0014 (5)
C12	0.0170 (5)	0.0125 (5)	0.0207 (5)	0.0004 (4)	0.0078 (4)	−0.0001 (4)
C13	0.0176 (5)	0.0154 (5)	0.0260 (5)	−0.0024 (5)	0.0047 (4)	−0.0003 (5)
C14	0.0187 (5)	0.0159 (6)	0.0252 (6)	0.0006 (4)	0.0064 (4)	0.0041 (5)
C15	0.0145 (5)	0.0276 (6)	0.0151 (5)	−0.0006 (5)	0.0007 (4)	−0.0026 (5)
C16	0.0234 (6)	0.0262 (7)	0.0210 (6)	0.0021 (5)	0.0044 (5)	−0.0042 (5)

### Geometric parameters (Å, °)

**Table:**

S1—C6	1.7824 (12)	C6—C7	1.3921 (18)
S1—C1	1.8465 (12)	C7—C8	1.386 (2)
O1—C1	1.4096 (13)	C7—H7	0.95
O1—C5	1.4298 (13)	C8—C9	1.383 (2)
O2—C15	1.3522 (14)	C8—H8	0.95
O2—C14	1.4460 (14)	C9—C10	1.384 (2)
O3—C15	1.2024 (16)	C9—H9	0.95
O4—C12	1.3596 (14)	C10—C11	1.3929 (19)
O4—C4	1.4527 (14)	C10—H10	0.95
O5—C12	1.1974 (15)	C11—H11	0.95
C1—C2	1.4975 (17)	C12—C13	1.4967 (17)
C1—H1	1	C13—H13A	0.98
C2—C3	1.3228 (19)	C13—H13B	0.98
C2—H2	0.95	C13—H13C	0.98
C3—C4	1.5046 (18)	C14—H14A	0.99
C3—H3	0.95	C14—H14B	0.99
C4—C5	1.5199 (16)	C15—C16	1.4980 (18)
C4—H4	1	C16—H16A	0.98
C5—C14	1.5069 (16)	C16—H16B	0.98
C5—H5	1	C16—H16C	0.98
C6—C11	1.3864 (18)		
			
C6—S1—C1	97.40 (5)	C9—C8—H8	119.8
C1—O1—C5	113.11 (8)	C7—C8—H8	119.8
C15—O2—C14	115.90 (9)	C8—C9—C10	119.91 (13)
C12—O4—C4	116.37 (9)	C8—C9—H9	120
O1—C1—C2	112.43 (9)	C10—C9—H9	120
O1—C1—S1	111.69 (8)	C9—C10—C11	120.14 (13)
C2—C1—S1	110.14 (8)	C9—C10—H10	119.9
O1—C1—H1	107.4	C11—C10—H10	119.9
C2—C1—H1	107.4	C6—C11—C10	119.78 (12)
S1—C1—H1	107.4	C6—C11—H11	120.1
C3—C2—C1	121.21 (11)	C10—C11—H11	120.1
C3—C2—H2	119.4	O5—C12—O4	122.84 (11)
C1—C2—H2	119.4	O5—C12—C13	126.21 (11)
C2—C3—C4	121.95 (11)	O4—C12—C13	110.94 (10)
C2—C3—H3	119	C12—C13—H13A	109.5
C4—C3—H3	119	C12—C13—H13B	109.5
O4—C4—C3	108.61 (10)	H13A—C13—H13B	109.5
O4—C4—C5	106.45 (9)	C12—C13—H13C	109.5
C3—C4—C5	109.66 (10)	H13A—C13—H13C	109.5
O4—C4—H4	110.7	H13B—C13—H13C	109.5
C3—C4—H4	110.7	O2—C14—C5	107.18 (9)
C5—C4—H4	110.7	O2—C14—H14A	110.3
O1—C5—C14	107.73 (9)	C5—C14—H14A	110.3
O1—C5—C4	107.84 (9)	O2—C14—H14B	110.3
C14—C5—C4	112.17 (10)	C5—C14—H14B	110.3
O1—C5—H5	109.7	H14A—C14—H14B	108.5
C14—C5—H5	109.7	O3—C15—O2	123.27 (12)
C4—C5—H5	109.7	O3—C15—C16	125.96 (11)
C11—C6—C7	120.04 (12)	O2—C15—C16	110.75 (11)
C11—C6—S1	120.02 (9)	C15—C16—H16A	109.5
C7—C6—S1	119.93 (10)	C15—C16—H16B	109.5
C8—C7—C6	119.72 (13)	H16A—C16—H16B	109.5
C8—C7—H7	120.1	C15—C16—H16C	109.5
C6—C7—H7	120.1	H16A—C16—H16C	109.5
C9—C8—C7	120.41 (13)	H16B—C16—H16C	109.5
			
C5—O1—C1—C2	−46.21 (12)	C1—S1—C6—C11	−90.11 (10)
C5—O1—C1—S1	78.20 (10)	C1—S1—C6—C7	89.10 (11)
C6—S1—C1—O1	68.09 (9)	C11—C6—C7—C8	0.25 (19)
C6—S1—C1—C2	−166.23 (8)	S1—C6—C7—C8	−178.96 (11)
O1—C1—C2—C3	7.92 (16)	C6—C7—C8—C9	−0.2 (2)
S1—C1—C2—C3	−117.34 (12)	C7—C8—C9—C10	−0.1 (2)
C1—C2—C3—C4	6.16 (19)	C8—C9—C10—C11	0.3 (2)
C12—O4—C4—C3	89.25 (12)	C7—C6—C11—C10	−0.06 (19)
C12—O4—C4—C5	−152.74 (10)	S1—C6—C11—C10	179.15 (10)
C2—C3—C4—O4	131.42 (12)	C9—C10—C11—C6	−0.2 (2)
C2—C3—C4—C5	15.47 (16)	C4—O4—C12—O5	4.37 (17)
C1—O1—C5—C14	−170.14 (9)	C4—O4—C12—C13	−175.44 (10)
C1—O1—C5—C4	68.59 (11)	C15—O2—C14—C5	158.61 (10)
O4—C4—C5—O1	−167.47 (9)	O1—C5—C14—O2	65.92 (11)
C3—C4—C5—O1	−50.15 (12)	C4—C5—C14—O2	−175.56 (9)
O4—C4—C5—C14	74.07 (12)	C14—O2—C15—O3	−1.62 (17)
C3—C4—C5—C14	−168.62 (10)	C14—O2—C15—C16	176.90 (10)

### Hydrogen-bond geometry (Å, °)

**Table:**

*D*—H···*A*	*D*—H	H···*A*	*D*···*A*	*D*—H···*A*
C5—H5···S1	1	2.86	3.2848 (12)	106
C13—H13B···O5^i^	0.98	2.44	3.3506 (15)	154

Symmetry codes: (i) *x*−1, *y*, *z*.

## References

[bb1] Allen, F. H. (2002). *Acta Cryst.* B**58**, 380–388. 10.1107/s0108768102003890 12037359

[bb2] Altomare, A., Burla, M. C., Camalli, M., Cascarano, G. L., Giacovazzo, C., Guagliardi, A., Moliterni, A. G. G., Polidori, G. & Spagna, R. (1999). *J. Appl. Cryst.* **32**, 115–119.

[bb3] Bracherro, M. P., Cabrera, E. F., Gomez, G. M. & Peredes, L. M. R. (1998). *Carbohydr. Res.* **308**, 181–190.

[bb4] Brandenburg, K. & Putz, H. (2005). *DIAMOND* Crystal Impact GbR, Bonn, Germany.

[bb5] Breton, G. W. J. (1997). *J. Org. Chem.* **62**, 8952–8954

[bb6] Bruker (2008). *SADABS*, *SAINT* and *XPREP* Bruker AXS Inc., Madison, Wisconsin, USA.

[bb7] Bruker (2011). *APEX2* Bruker AXS Inc., Madison, Wisconsin, USA.

[bb8] Chambers, D. J., Evans, G. R. & Fairbanks, A. (2005). *Tetrahedron Asymmetry*, **16**, 45–55.

[bb9] Cremer, D. & Pople, J. A. (1975). *J. Am. Chem. Soc.* **97**, 1354–1358.

[bb10] Danishefsky, S. J. & Bilodeau, M. T. (1996). *Angew. Chem. Int. Ed. Engl.* **35**, 1380–1419.

[bb11] Domon, D., Fujiwara, K., Ohtaniuchi, Y., Takezawa, A., Takeda, S., Kawasaki, H., Murai, A., Kawai, H. & Suzuki, T. (2005). *Tetrahedron Lett.* **46**, 8279–8283.

[bb12] Dorgan, B. J. & Jackson, R. F. W. (1996). *Synlett*, pp. 859–861.

[bb13] Farrugia, L. J. (1999). *J. Appl. Cryst.* **32**, 837–838.

[bb14] Ferrier, R. J. & Prasad, N. J. (1969). *J. Chem. Soc.* pp. 570–575.

[bb15] Flack, H. D. (1983). *Acta Cryst.* A**39**, 876–881.

[bb16] Griffith, D. A. & Danishefsky, S. J. (1991). *J. Am. Chem. Soc.* **113**, 5863–5864.

[bb17] Halcomb, R. H., Boyer, S. H., Wittman, M. D., Olson, S. H., Denhart, D. J., Liu, K. K. C. & Danishefsky, S. J. (1995). *J. Am. Chem. Soc.* **117**, 5720–5749.

[bb18] Kinfe, H. H., Mebrahtu, F. M. & Sithole, K. (2011). *Carbohydr. Res.* doi:10.1016/j.carres.2011.08.023. 10.1016/j.carres.2011.08.023 22000161

[bb19] López, J. C., Gómez, A. M., Valverde, S. & Fraser-Reid, B. (1995). *J. Org. Chem.* **60**, 3851–3858.

[bb20] Minuth, T. & Boysen, M. M. K. (2009). *Org. Lett.* **11**, 4212–4215. 10.1021/ol901579g 19691311

[bb21] Sheldrick, G. M. (2008). *Acta Cryst.* A**64**, 112–122. 10.1107/S0108767307043930 18156677

[bb22] Yadav, J. S., Reddy, B. V. S. & Chand, P. K. (2001). *Tetrahedron Lett.* **42**, 4057–4059.

